# Supplementary figures and images for: Circadian regulation of macromolecular complex turnover and proteome renewal
Source: EMBO J. 2024 May 22;43(13):2813–33. doi: 10.1038/s44318-024-00121-5 (PMC11217436; doi:10.1038/s44318-024-00121-5)

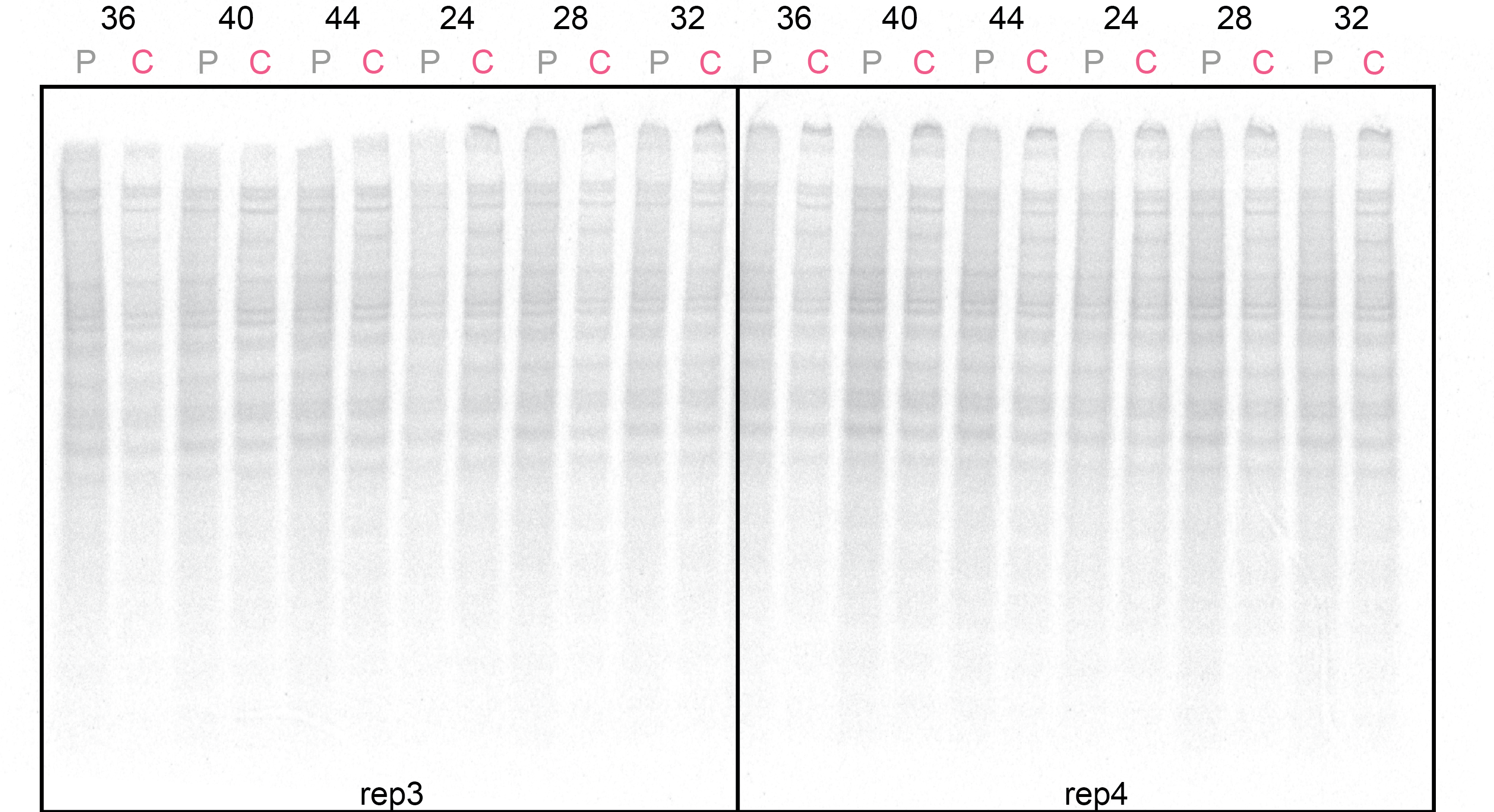

Supplement: Supplementary file 5 — Source data Fig. 1 [file 44318_2024_121_MOESM5_ESM.zip › figure 1/A/35S incorporation, replicates 3 and 4.png]

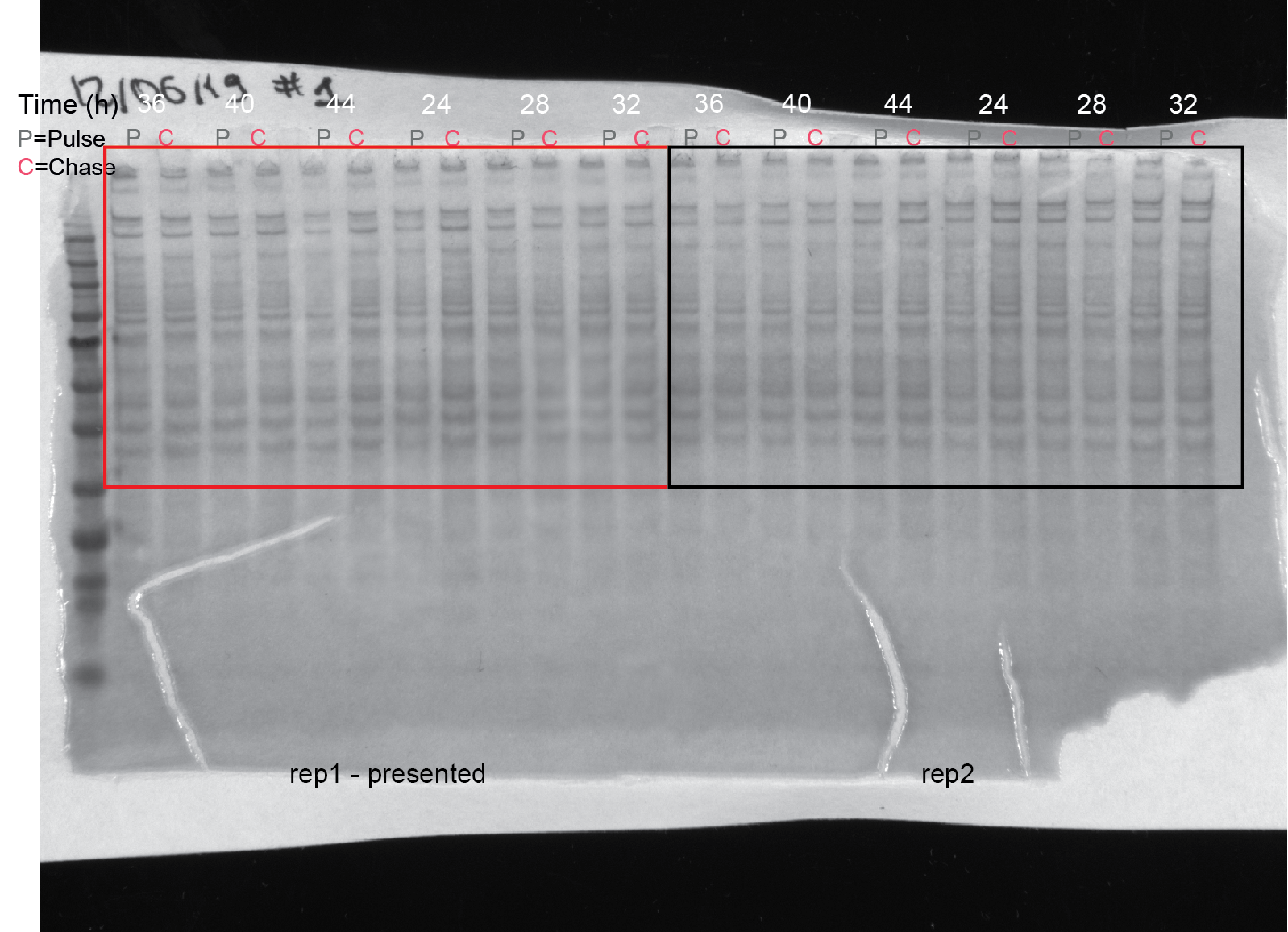

Supplement: Supplementary file 5 — Source data Fig. 1 [file 44318_2024_121_MOESM5_ESM.zip › figure 1/A/Coomassie gel, replicates 1 and 2.png]

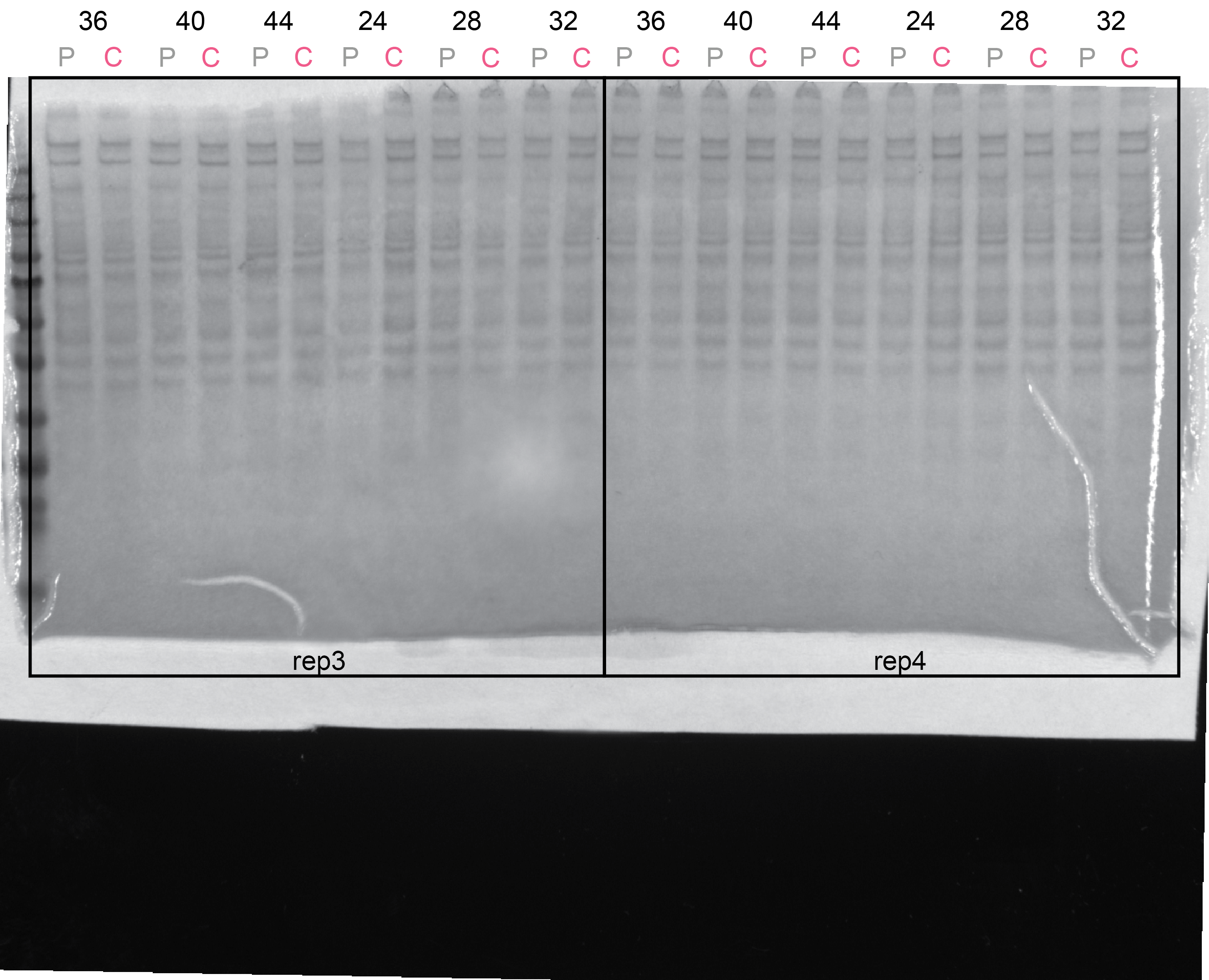

Supplement: Supplementary file 5 — Source data Fig. 1 [file 44318_2024_121_MOESM5_ESM.zip › figure 1/A/Coomassie gel, replicates 3 and 4.png]

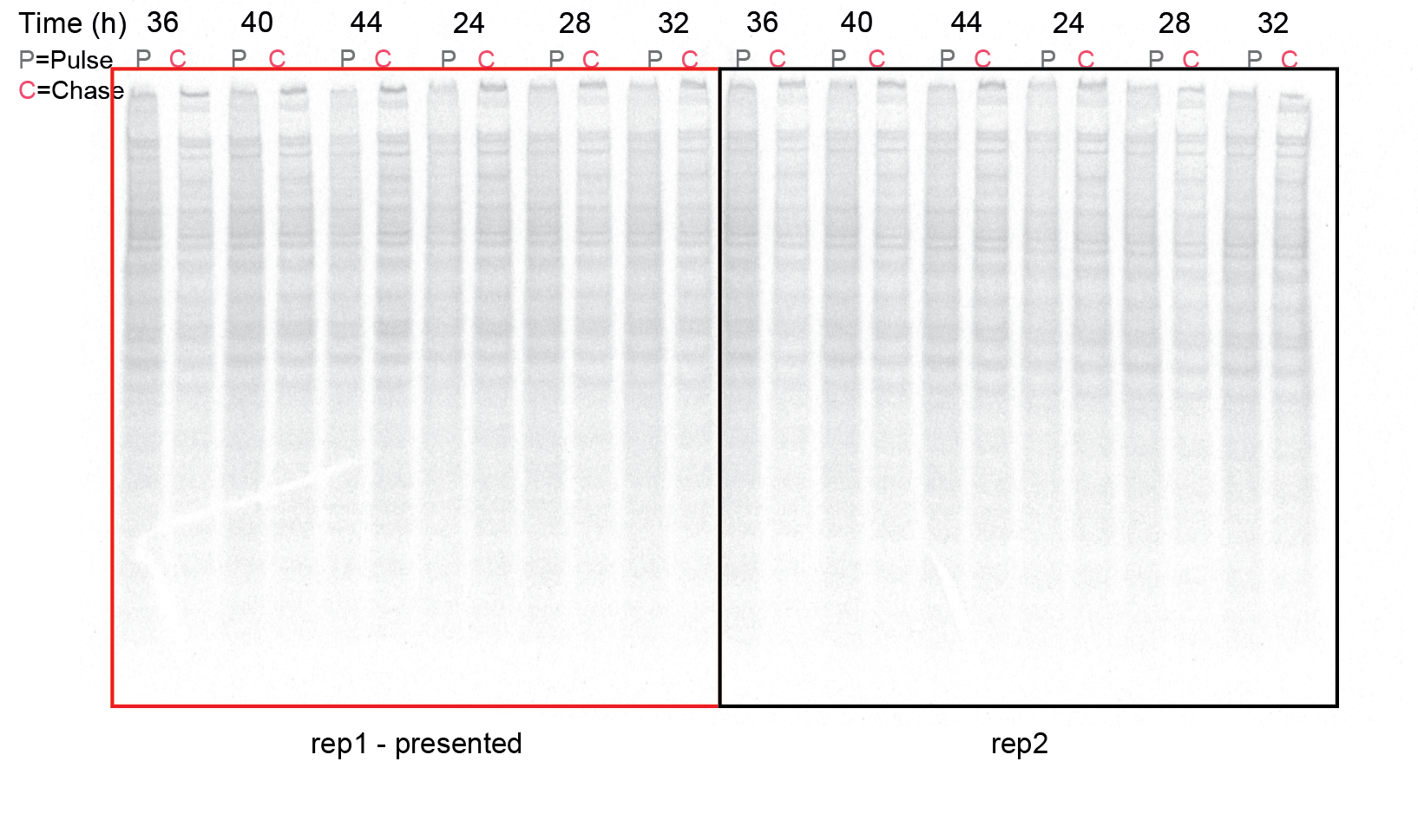

Supplement: Supplementary file 5 — Source data Fig. 1 [file 44318_2024_121_MOESM5_ESM.zip › figure 1/A/35S incorporation, replicates 1 and 2.png]

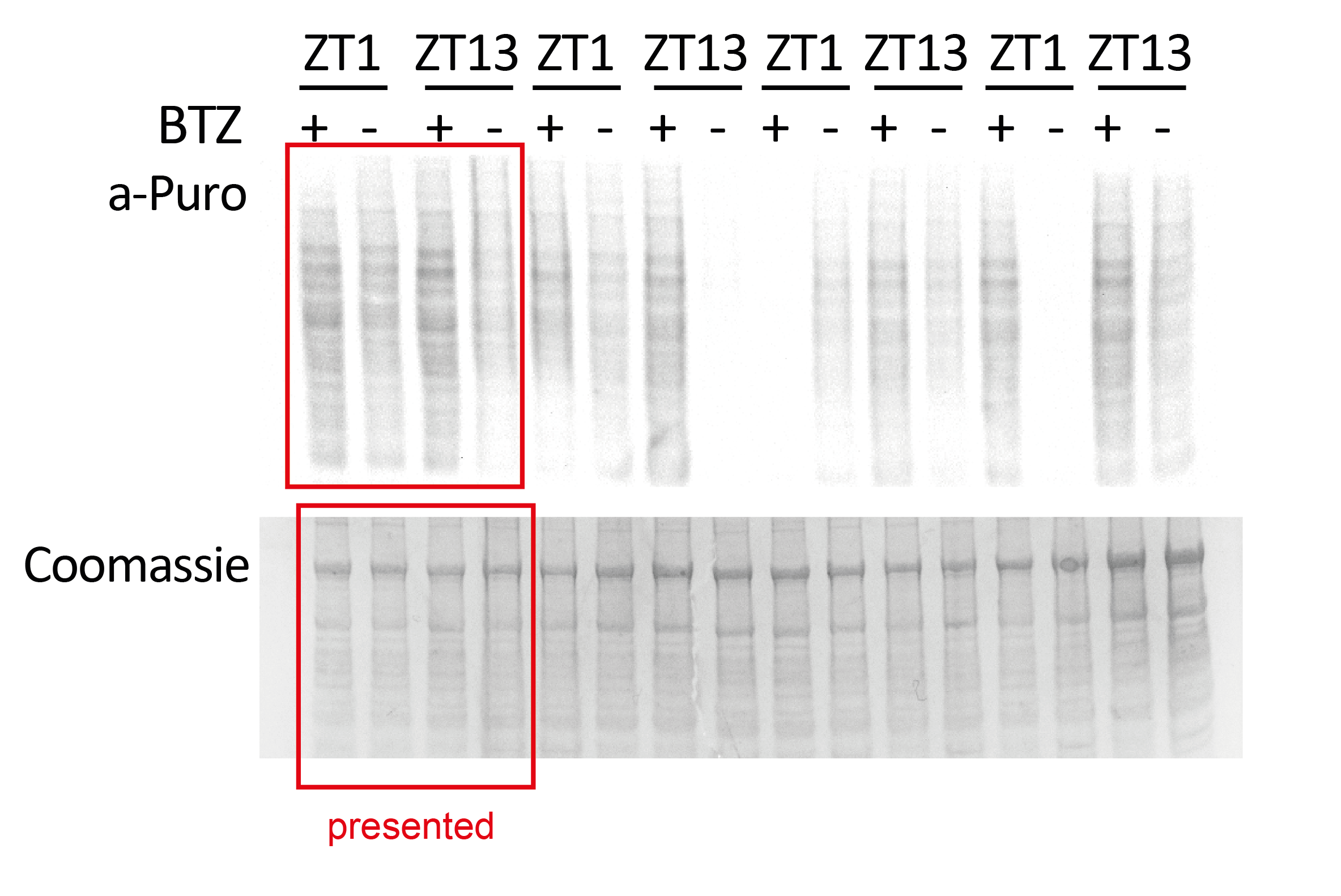

Supplement: Supplementary file 5 — Source data Fig. 1 [file 44318_2024_121_MOESM5_ESM.zip › figure 1/F/puro tissue blots 1F.png]

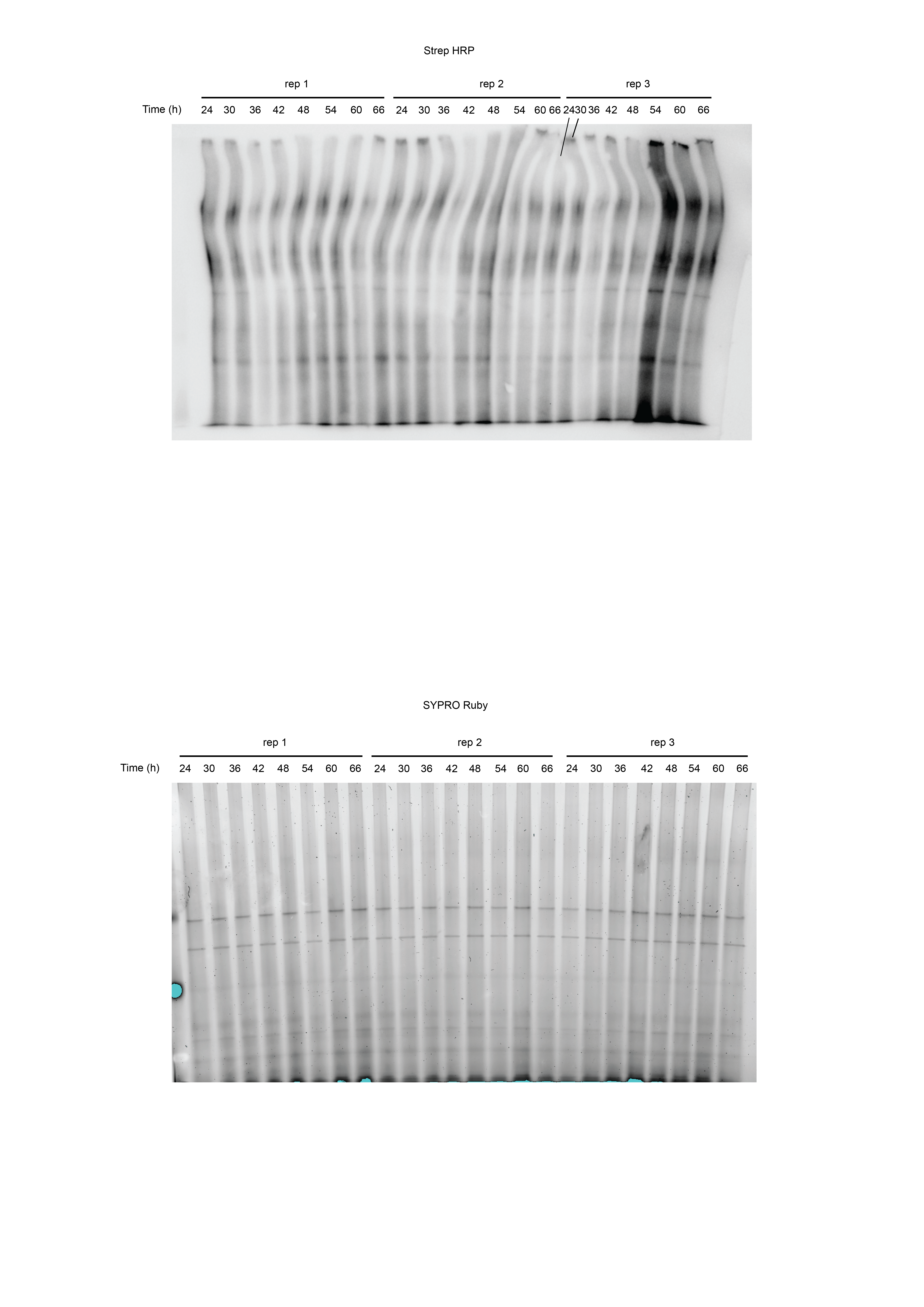

Supplement: Supplementary file 7 — Source data Fig. 3 [file 44318_2024_121_MOESM7_ESM.zip › figure 3/H/strep and sypro ruby gels.png]
